# Structural Characterization of the *Chaetomium thermophilum* TREX-2 Complex and its Interaction with the mRNA Nuclear Export Factor Mex67:Mtr2

**DOI:** 10.1016/j.str.2015.05.002

**Published:** 2015-07-07

**Authors:** Lyudmila Dimitrova, Eugene Valkov, Shintaro Aibara, Dirk Flemming, Stephen H. McLaughlin, Ed Hurt, Murray Stewart

**Affiliations:** 1 Medical Research Council Laboratory of Molecular Biology, Francis Crick Avenue, Cambridge Biomedical Campus, Cambridge CB2 0QH, UK; 2 Biochemie-Zentrum der Universität Heidelberg, INF328, 69120 Heidelberg, Germany

## Abstract

The TREX-2 complex integrates mRNA nuclear export into the gene expression pathway and is based on a Sac3 scaffold to which Thp1, Sem1, Sus1, and Cdc31 bind. TREX-2 also binds the mRNA nuclear export factor, Mex67:Mtr2, through the Sac3 N-terminal region (Sac3N). Here, we characterize *Chaetomium thermophilum* TREX-2, show that the in vitro reconstituted complex has an annular structure, and define the structural basis for interactions between Sac3, Sus1, Cdc31, and Mex67:Mtr2. Crystal structures show that the binding of *C. thermophilum* Sac3N to the Mex67 NTF2-like domain (Mex67^NTF2L^) is mediated primarily through phenylalanine residues present in a series of repeating sequence motifs that resemble those seen in many nucleoporins, and Mlp1 also binds Mex67:Mtr2 using a similar motif. Deletion of Sac3N generated growth and mRNA export defects in *Saccharomyces cerevisiae*, and we propose TREX-2 and Mlp1 function to facilitate export by concentrating mature messenger ribonucleoparticles at the nuclear pore entrance.

## Highlights

• *Chaetomium thermophilum* TREX-2 has an annular structure resembling the letter Q
• Structure of interfaces between TREX-2 components Sac3, Sus1, and Cdc31 defined
• Sac3N phenylalanines dominate *C. thermophilum* TREX-2 binding to Mex67 NTF2L domain
• TREX-2 facilitates mRNA export by concentrating mature mRNPs at nuclear pores

## Teaser

Dimitrova et al. show that in *Chaetomium thermophilum*, the Sac3:Thp1:Sem1:Sus1:Cdc31 TREX-2 complex that integrates mRNA nuclear export into the gene expression pathway, has an annular structure and binds mRNA nuclear export factor Mex67:Mtr2 primarily through phenylalanine-containing motifs in Sac3N, thereby facilitating mRNA export by concentrating mature mRNPs at nuclear pores.

## Introduction

The export of mRNA from the nucleus to the cytoplasm is a crucial step in the gene expression pathway in eukaryotes, enabling the genetic message encoded in the genome to be ultimately used for protein synthesis by ribosomes. Export of mRNA requires the assembly of export competent ribonucleoparticles (RNPs), a process that is tightly integrated with transcription and pre-mRNA maturation (Strässer et al., 2002; González-Aguilera et al., 2008). Once assembled, RNPs exit the nucleus through nuclear pore complexes (NPCs), 8-fold symmetric, supramolecular assemblies embedded in the nuclear envelope that are composed of proteins called nucleoporins. Whereas many nucleoporins contribute to the cylindrical NPC scaffold, others that contain regions rich in Phe-Gly (FG) sequence motifs fill the central channel of the NPC and generate a selective barrier (reviewed by Grossman et al., 2012). Although some FG nucleoporins are distributed symmetrically across the NPC, others are distributed asymmetrically and are found at either the nuclear or cytoplasmic face (Terry and Wente, 2007). Transport through NPCs is facilitated by transport factors that bind a macromolecular cargo in one compartment and release it in the other, and which can overcome the barrier function through interactions with the nucleoporin FG sequence motifs. NPCs also have peripheral appendages on their nuclear and cytoplasmic faces. A basket-like structure composed of eight filaments attached at a distal ring is located at the nuclear face and contains Mlp1 and Mlp2 (TPR in higher eukaryotes) together with several nucleoporins such as Nup1 (Rout et al., 2000). In addition to contributing to chromatin maintenance and transcription, nuclear basket components also facilitate mRNA export by docking mRNPs to the NPC through an interaction with the mRNA binding protein Nab2 (Green et al., 2003; Grant et al., 2008).

Whereas protein transport is generally facilitated by karyopherin-β family proteins (reviewed by Chook and Süel, 2011), mRNA export in yeast is mediated primarily by the Mex67:Mtr2 complex or in metazoans by NXF1:NXT1 (Segref et al., 1997; Braun et al., 2001). It is thought that after Mex67:Mtr2 is recruited to an RNP, it facilitates translocation through NPCs using serial transient, low-affinity interactions with nucleoporin FG repeats (Strässer et al., 2000). Mex67 is a modular protein containing four domains: RNA-recognition motif (RRM), leucine-rich repeat (LRR), nuclear transport factor 2-like (NTF2L), and ubiquitin-associated (UBA). The N-terminal RRM, LRR, and NTF2L domains are thought to recognize RNA, whereas the NTF2L domain binds Mtr2 and, together with the UBA domain, provides low-affinity binding sites for nucleoporin FG repeats (Kang et al., 1999; Liker et al., 2000; Fribourg et al., 2001; Bachi et al., 2000; Grant et al., 2002, 2003; Aibara et al., 2015).

**Figure 1:**
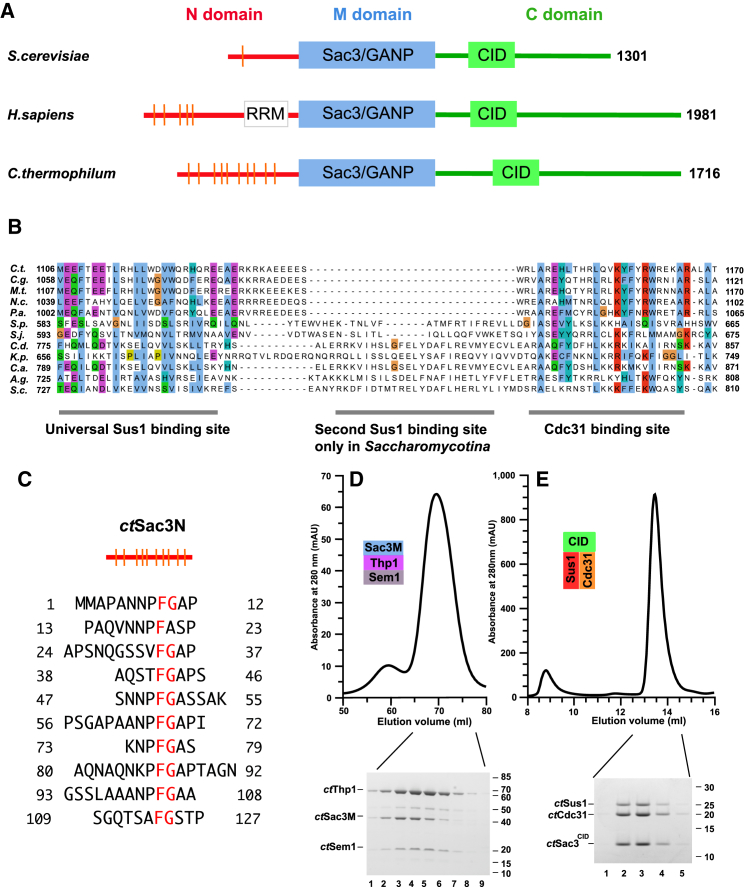
Identification and Characterization of *ct*TREX-2 Components (A) Schematic illustration of the structure of Sac3 from different organisms. Sac3 has an N-terminal domain of variable length (red), a conserved middle domain (blue) that interacts with Thp1 and Sem1, and a C-terminal domain (green) that contains a conserved CID motif. The N-terminal domains of human and *C. thermophilum* Sac3 have distinctive FG repeats (vertical lines). See also Figure S1. (B) Putative Sus1 binding domains in Sac3 sequences. (C) Repeating FG sequence motifs in *ct*Sac3N. (D) Size-exclusion chromatography of the *ct*Thp1:Sem1:Sac3^M^ complex reconstituted in vitro. (E) Tripartite *ct*Sac3^CID^:Sus1:Cdc31 complex. The CID region of *ct*Sac3 was tagged with GST-TEV and co-expressed with Sus1 and Cdc31. GSH purification was followed by TEV cleavage and size-exclusion chromatography. Fractions were analyzed by SDS-PAGE.

Transcription export complex 2 (TREX-2) contributes to both mRNA nuclear export and its integration with the nuclear steps of the gene expression pathway (Rodriguez-Navarro et al., 2004; González-Aguilera et al., 2008). TREX-2 is based on a Sac3 scaffold (Figure 1A) to which Thp1, Sem1, Sus1, and Cdc31 bind, and is localized primarily at NPCs through interactions with proteins located on the nucleoplasmic face, including Nup1 (Fischer et al., 2002, 2004; Faza et al., 2009; Jani et al., 2009, 2014; Ellisdon and Stewart, 2012). Mutations in TREX-2 components generate mRNA nuclear export defects and frequently also growth defects. TREX-2 also interacts with Mex67:Mtr2 and mutations of *MEX67* and *MTR2* are synthetically lethal in *Saccharomyces cerevisiae* when combined with mutations in *SAC3* (Fischer et al., 2002, 2004). Previous work has indicated that Mex67:Mtr2 binds primarily to the N-terminal region of Sac3 (Fischer et al., 2002). In yeast, TREX-2 also facilitates the localization of many actively transcribing genes, such as *GAL1*, to NPCs, which in turn facilitates the removal of repression mediated by de-ubiquitinylation by Ulp1 (Texari et al., 2013) and, through interactions between TREX-2 and the SAGA complex, can couple transcription, processing, and polyadenylation with the export of mature mRNAs to the cytoplasm (Rodriguez-Navarro et al., 2004; Köhler et al., 2008).

Although crystal structures have been obtained for parts of the *Saccharomyces* TREX-2 complex, such as Sac3^CID^ bound to Sus1 and Cdc31 (Jani et al., 2009, 2014) or Sac3^M^ bound to Thp1 and Sem1 (Ellisdon et al., 2012), it has been more difficult to obtain structural information about the complete complex and its interactions with Mex67:Mtr2. Here, we exploit the thermophilic fungus *Chaetomium thermophilum* to show that TREX-2 has an annular, ring-like conformation, and also define how it interacts with Mex67:Mtr2. We show that *C. thermophilum* Sac3N contains ten repeating FG sequence motifs that bind Mex67:Mtr2, but not karyopherin family nuclear transport factors, indicating that the Sac3N motifs are distinct from those in FG nucleoporins. The Sac3N motifs bind primarily to the Mex67 NTF2L domain, and crystal structures of complexes between Mex67^NTF2L^:Mtr2 and Sac3N peptides identify a central role of the motif's Phe residue binding into a hydrophobic pocket on Mex67^NTF2L^. Similar repeating sequence motifs were also identified in the nuclear basket component Mlp1, and these motifs appear to interact specifically with Mex67:Mtr2, in a manner similar to that for Sac3N.

## Results

### Identification and Characterization of *C. thermophilum* TREX-2 Components

**Figure 2:**
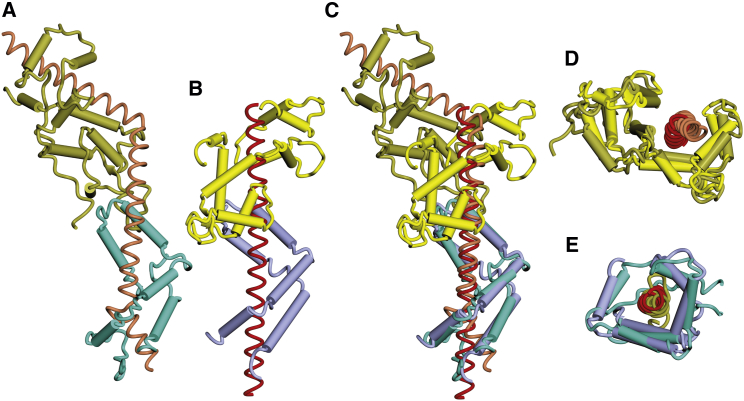
*C. thermophilum* TREX-2 CID Region Complex (A) Crystal structure at 3.3-Å resolution of the *C. thermophilum* TREX-2 CID region in which Cdc31 (gold) and a single Sus1 chain (cyan) wrap around the Sac3 α helix (pink) that shows a distinct bend rather than the primarily straight conformation in other species (Jani et al., 2009, 2012). (B) Structure of the corresponding *S. cerevisiae* complex (PDB: 3FWB) with Sac3 colored red, Cdc31 yellow, and Sus1 sky blue. (C) Superposition of the *C. thermophilum* and *S. cerevisiae* complexes. (D and E) Superposition of the Cdc31 (D) and Sus1 (E) chains showing the conservation of each interaction. See also Figure S2.

The *Saccharomyces* TREX-2 complex consists of a Sac3 scaffold (Figure 1A) onto which Sus1 and Cdc31 bind at the CID region and Sem1 and Thp1 bind at the M region (Rodriguez-Navarro et al., 2004; Jani et al., 2009). A spectrum of methods was used to identify components of the *C. thermophilum* (*ct*) complex (Figure S1). BLAST searches of the *C. thermophilum* genome using the *Aspergillus nidulans* sequence (Osmani et al., 2006) identified Sac3 (XP_006696971), which retained the characteristic conserved M domain with a PCI fold and a conserved CID motif with putative Cdc31 (residues 1,140–1,165) and Sus1 (residues 1,091–1,120) binding sites. Compared with its *S. cerevisiae* and human homologs, the *ct*Sac3 N-terminal region (Sac3N) had more prominent FG repeats (Figures 1A and 1B; Figure S1A). However, as noted by Fischer et al. (2002), the motifs in Sac3N sequences, like those of many FG motifs in nucleoporins, are not strongly conserved between species. Probing with the *S. cerevisiae* sequences identified *ct*Cdc31 (XP_006694724), *ct*Sem1 (CTHT_0037380), and two putative Thp1 homologs (CTHT_0034350 and CTHT_0056910; Figure S1D), but only CTHT_0034350 formed a tripartite complex in vitro with the *ct*Sac3M domain (Figure 1C). Sus1 (CTHT_0027270) was identified by probing *C. thermophilum* and other *Pezyzomycotina* genome sequences for small open reading frames with a predicted secondary structure based on the five α helices that characterize yeast and human Sus1 (Figure S1E). Bacterially expressed *ct*Sus1 formed a tripartite complex with *ct*Sac3^CID^ and *ct*Cdc31 (Figure 1D), and the crystal structure of this complex confirmed that the appropriate *Chaetomium* genes had been identified. Crystalline plates of the *C. thermophilum* Sac3^CID^:Sus1:Cdc31 complex were obtained that diffracted to 3.3 Å resolution, and molecular replacement produced clear and well-defined electron density maps. After refinement, the model (Figure 2A) had a *R*_work_/*R*_free_ value of 21.8%/27.7% and excellent geometry (Table 1). Although its overall fold was similar to its *S. cerevisiae* homolog, the *C. thermophilum* Sac3^CID^:Cdc31:Sus1 complex had only a single Sus1 chain, whereas in the corresponding *S. cerevisiae* and GANP structures Sac3 bound two Sus1 chains, although only one of these chains appeared to be important functionally (Jani et al., 2009, 2012). In the *C. thermophilum* complex, the Sac3 helix also had a “kink” following residue 1,137 (Figure 2), possibly as a consequence of the greater distance between the Cdc31 and Sus1 chains. The overall folds of *ct*Cdc31 and *ct*Sus1 were strongly conserved with their *S. cerevisiae* counterparts, with root-mean-square deviations (RMSDs) of 2.56 Å over 88 C-α atoms for Sus1 and 3.10 Å over 144 C-α atoms for Cdc31. The helical elements of the folds of both proteins were fully conserved, with differences concentrated in the loops joining the helices. Density for *ct*Sus1 residues 99–139, as well as its substantially longer N/C-terminal extensions, was not present, consistent with these regions being disordered. Importantly, the extensive interaction interfaces between Sac3 and both Cdc31 and Sus1 were preserved. For example, *ct*Sac3 Trp1161 was analogous to *sc*Sac3 Trp802, where it is a central feature of the Sac3-Cdc31 interaction and is buried in a hydrophobic cavity in the Cdc31 C-terminal domain.

### In Vitro Reconstituted ctTREX-2 Has a Ring-like Structure

**Figure 3:**
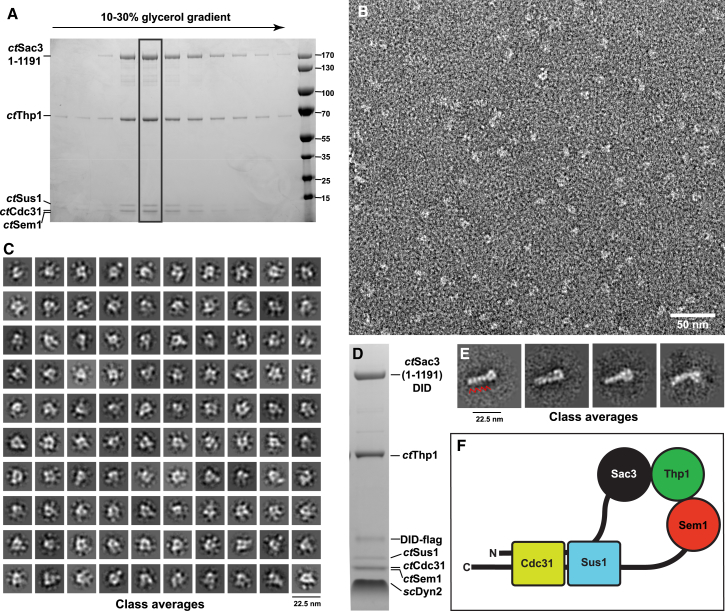
Reconstitution of *C. thermophilum* TREX-2 (A) Purification of in vitro reconstituted *ct*TREX-2 based on Sac3 residues 1–1,191 using a 10%–30% glycerol gradient. Gradient fractions were analyzed by 4%–12% gradient SDS-PAGE gel and stained with Coomassie. (B) Electron micrograph of *ct*TREX-2 (from the boxed fraction in Figure 1A) negatively stained with uranyl formate. Fields contained particles that were generally 10–11 nm in diameter and which frequently had an annular appearance. (C) Class averages derived from analysis of 27,189 single particles. Many have an annular, ring-like appearance and frequently also have an additional small protuberance so that they resemble the letter “Q.” (D) SDS-PAGE of Dyn2-labeled TREX-2-DID complex purified by glycerol gradient centrifugation. (E) Class averages of electron micrographs of negatively stained Dyn2-labeled TREX-2-DID showed characteristic 20-nm rods formed by the Dyn2-bound DID label (red arrowheads in first panel) that had a 10-nm diameter ring at one end. (F) Highly schematic illustration of how an intermolecular interaction between the Sac3 N-terminal and CID regions could generate the annular TREX-2 particles observed on electron micrographs.

Because full-length *ct*Sac3 could not be expressed in bacteria and only very limited amounts could be obtained by expression in *S. cerevisiae*, a C-terminally truncated Sac3 construct (residues 1–1,191, containing the Sac3N and Sac3M regions, and most of the C-terminal domain including the CID region) was used to reconstitute the *ct*TREX-2 complex. Truncated *ct*Sac3 with an N-terminal TEV-cleavable protein A tag and a C-terminal FLAG tag was co-expressed with *ct*Sus1 and *ct*Cdc31 in *S. cerevisiae* and affinity purified on immunoglobulin G (IgG) beads, after which purified *ct*Thp1:Sem1 complex was added. After binding and washing, it was eluted by TEV protease cleavage and affinity purified on FLAG beads. The second affinity step eliminated C-terminal degradation products of Sac3 and produced a stoichiometric Sac3^1−1191^:Thp1:Sus1:Cdc31:Sem1 complex that was further purified using a 10%–30% glycerol density gradient (Figure 3A).

Electron micrographs of the reconstituted *ct*TREX-2 complex negatively stained with uranyl formate (Figure 3B) showed fields that contained particles of ∼10–11 nm diameter, many of which had a characteristic ring-like appearance, containing two or three dots, often with a small protuberance so that they resembled the uppercase letter Q. This appearance was particularly marked when single particle methods were used to classify 27,189 particles to generate class averages such as those shown in Figure 3C, in which a Q-like appearance was frequently generated. However, there was considerable variation between the different classes, consistent with TREX-2 being flexible. This flexibility, combined with a marked preferred orientation of the particles on the grid, frustrated generation of a three-dimensional model of the complex. Unfortunately, employing the Grafix procedure to improve the sample generated dimers with a figure-of-eight appearance. The annular appearance was also seen when a dynein light chain-interacting domain (DID) label (Flemming et al., 2010) was attached to the C terminus of *ct*Sac3. The DID label has six QT recognition motifs (based on 12-residue peptides) that have a high affinity for *S. cerevisiae* dynein light chain dimers (Dyn2) and on electron micrographs appears as a 20-nm rod that is easily visualized in negatively stained material. Class averages (Figure 3E) of negatively stained *ct*TREX2 containing DID-labeled Sac3 (Figure 3D) showed these rods to which a 10-nm diameter ring was attached at one end, confirming that the rings genuinely contained TREX-2. The small protuberance that gave rise to a Q-like appearance in the unlabeled TREX-2 micrographs was not observed in class averages of the labeled material indicating that this feature may be associated with the Sac3 C-terminal region.

One way in which Q-shaped *C. thermophilum* TREX-2 particles could be generated would be if regions near the Sac3 N terminus were to interact with the CID region. In *S. cerevisiae* the TREX-2 CID region binds a Phe-rich motif in Nup1 in a cavity formed between Sac3 and Sus1 (Jani et al., 2014), and the hydrophobic residues that are crucial in forming this interface were generally conserved in the crystal structure of the *C. thermophilum* TREX-2 CID region (Figure 2E), consistent with its having the potential to bind analogous Phe-rich motifs. To evaluate whether the *ct*Sac3 CID region could potentially interact with the Sac3N FG region, their interaction was analyzed by surface plasmon resonance (SPR) (Figure S2). Although the overall equilibrium dissociation constant was 2.2 μM, both the on-rate (235 M^-1^ s^−1^) and off-rate (5.2 × 10^−4^ s^−1^) were very slow. However, in the context of the complete TREX-2 complex, the slow association rate constant may be overcome due to high local concentration of the interacting regions.

Previous studies have generated structures for both the *S. cerevisiae* CID region (Jani et al., 2009) and the Sac3M region complexed with Thp1 and Sem1 (Ellisdon et al., 2012), showing that both form relatively compact globular regions, as does the *C. thermophilum* CID region (Figure 2). Therefore, the SPR and electron microscopy data are consistent with TREX-2 forming annular structures through a Sac3N:CID interaction, although the limited resolution obtained precluded assignment of individual chains to specific regions.

### Sac3N Binds Primarily the Mex67 NTF2-like Domain Complexed with Mtr2

**Figure 4:**
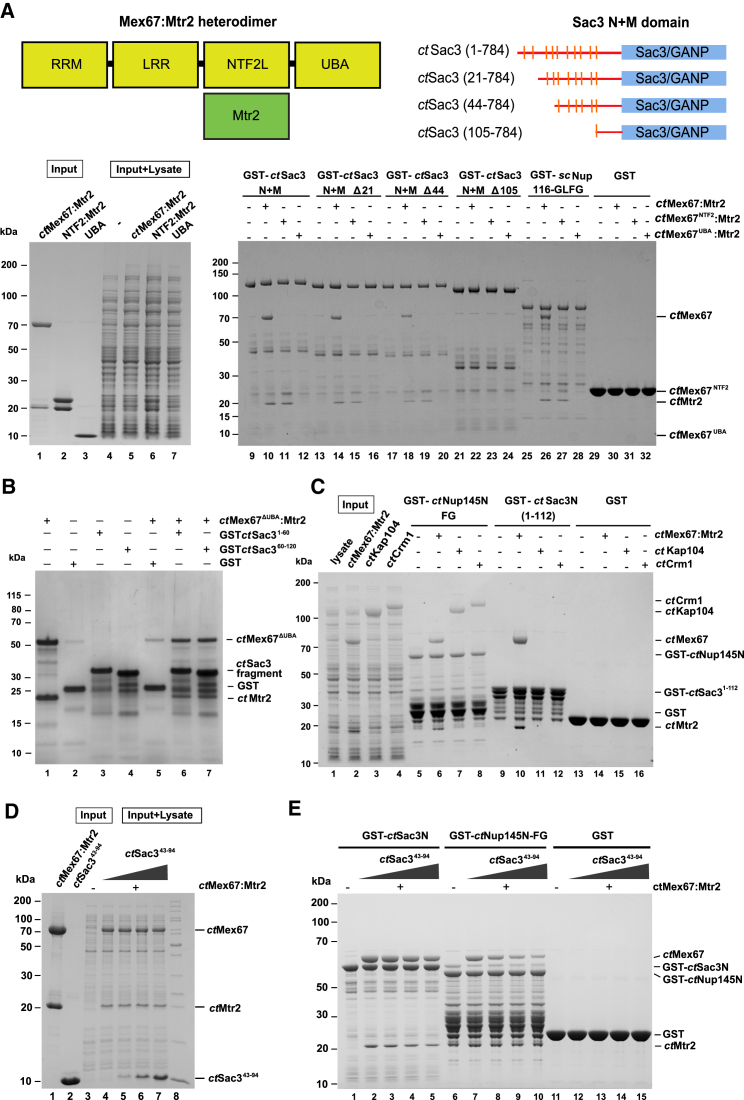
*ct*Sac3N Interactions with *ct*Mex67:Mtr2, *ct*Kap104, and *ct*CRM1/Xpo1 (A) *ct*Mex67:Mtr2 is recruited to the N-terminal region of *ct*Sac3 that contains ten FG sequence motifs (residues 1–105). The Sac3 N-terminal truncations (upper right) were immobilized on GSH beads before addition of Mex67:Mtr2 or different truncations corresponding to its different functional domains (upper left). The purified proteins in lanes 2–4 were mixed with *E. coli* lysate (lanes 6–8) prior to addition to the immobilized baits. After incubation, washing, and elution with SDS loading buffer, the proteins were analyzed by SDS-PAGE with Coomassie staining. (B) Both the first five (residues 1–60) and second five FG motifs (residues 60–120) in *ct*Sac3N bind to *ct*Mex67:Mtr2, consistent with individual FG motifs having similar affinities. (C) The *ct*Sac3N region provides a specific recruiting platform for Mex67:Mtr2. In vitro binding assay of *ct*Mex67:Mtr2, *ct*Kap104 and *ct*Crm1 pre-mixed with *E. coli* lysate (lanes 2, 3, and 4, respectively) to *ct*Nup145N (lanes 5–8) and *ct*Sac3N (residues 1–112). (D and E) Competition assay indicating that the binding sites for Sac3N and nucleoporin FG repeats on Mex67:Mtr2 overlap. The inputs are presented in (D). The same concentration of Mex67:Mtr2 complex was mixed with *E. coli* lysate and increasing concentration of Sac3N^43−94^ (D, lanes 4–7). The mixture was added to GST-*ct*Sac3N^1−112^ (E), which contains ten sequential FG motifs (lanes 1–5), GST-*ct*Nup145 (lanes 6–10), and GST (lanes 10–15) immobilized on GSH beads. Samples were incubated at 4°C for 1 hr, washed, eluted with sample buffer, and analyzed by SDS-PAGE. See also Figure S4.

In *S. cerevisiae*, TREX-2 interacts with Mex67:Mtr2, and it has been suggested that this interaction could be mediated by degenerate FG repeats in the Sac3N region (Fischer et al., 2002); thus, because *ct*Sac3N contains prominent FG repeats (Figure 1B), it was an attractive model in which to study this interaction. Glutathione *S*-transferase (GST) pull-down assays with a range of N-terminal truncations of *ct*Sac3 (Figure 4A) indicated that the *ct*Sac3N FG repeats (residues 1–105) were sufficient for binding *ct*Mex67:Mtr2. Because both the NTF2-like and UBA domains of Mex67 interact with FG repeats (Kang et al., 1999; Liker et al., 2000; Fribourg et al., 2001; Bachi et al., 2000; Grant et al., 2002, 2003), a range of Mex67 constructs containing different domains were tested for binding to *ct*Sac3 N. In vitro purified *ct*Mex67:Mtr2, *ct*Mex67^NTF2^:Mtr2, and *ct*Mex67^UBA^ were pre-mixed with *Escherichia coli* lysate and added to different truncations of Sac3 immobilized on glutathione (GSH) beads. These results indicated that Sac3N interacted with Mex67:Mtr2 primarily through its NTF2-like domain (Figure 4A). Because the FG sequence motif was strongly conserved between the different *ct*Sac3N motifs, it seemed likely that they would probably have comparable affinities for Mex67:Mtr2. As shown in Figure 4B, Mex67^NTF2L^:Mtr2 bound equally well to the first five FG repeats and last five FG repeats, consistent with there not being a single repeat that had a markedly higher affinity.

Although many nuclear transport factors bind to nucleoporins that contain analogous FG repeats (based on motifs such as GLFG or FxFG), only *ct*Mex67:Mtr2 bound to GST-tagged *ct*Sac3N in pull-down assays, whereas no binding was observed for karyopherins such as *ct*CRM1 (Xpo1) and *ct*Kap104, although both *ct*Mex67:Mtr2 and the karyopherins bound to the FG nucleoporin Nup145 in these assays (Figure 4C). Thus, although the *ct*Sac3 N-terminal region contained repeating sequence motifs that contained an FG sequence in their core, these motifs appeared to be distinct from those found in FG nucleoporins, to which nuclear transport factors bind as they transit through NPCs.

Interactions between FG repeats and nuclear transport receptors are generally weak, so the observation that the *ct*Sac3^NM^:Mex67:Mtr2:Thp1:Sem1 complex remained intact during gel filtration (Figure S3) indicated that Sac3N might bind Mex67:Mtr2 more strongly. The kinetics of the interaction between *ct*Mex67:Mtr2 and both *ct*Sac3N and an FG-region fragment of *ct*Nup145 (residues 213–567) were assayed using SPR (Table 2; Figure S4), and both fitted well to double exponentials for association and dissociation phases, with the Sac3N interaction having *K*_D_s of 39 and 148 nM. The *K*_D_s for GST-Nup145 were more similar (*K*_D1_ = 238 nM, *K*_D2_ = 264 nM) but, although the off-rates were of the same order as those observed with GST-Sac3N, the first association rate was much slower, resulting in a weaker affinity (Table 2; Figure S4). The poor solubility of *ct*Sac3N and *ct*Nup145 peptides also precluded the determination of binding constants using isothermal calorimetry. However, although the absolute values of affinities determined by SPR using GST fusions can sometimes be anomalous, the relative values obtained for GST-Sac3N and GST-Nup145 indicate that the binding of *ct*Mex67:Mtr2 to *ct*Sac3N FG motifs was stronger than to classic nucleoporin FG repeats. A competition assay indicated that FG nucleoporins and Sac3N bind to overlapping sites on Mex67:Mtr2. Because the affinity of Sac3N for Mex67:Mtr2 was higher than Nup145N, increasing amounts of a Sac3N fragment (residues 43–94, containing four FG motifs) together with *E. coli* lysate (Figure 4D lanes 4–7) were mixed with equal amounts of Mex67:Mtr2 and added to GST-Nup145 immobilized on GSH beads (Figure 4E, lanes 6–10). The amount of *ct*Mex67:Mtr2 bound to the beads decreased as progressively higher amounts of the *ct*Sac3N fragment were added, consistent with the binding sites on Mex67:Mtr2 for overlapping of both Nup145N and Sac3N.

### Structural Basis for the Interaction of ctMex67:Mtr2 with ctSac3N

A spectrum of different 20-residue peptides derived from *ct*Sac3N was used in extensive crystallization trials with the *ct*Mex67^NTF2L^:Mtr2 complex. Diffraction quality crystals were obtained with *ct*Sac3^1−20^ and *ct*Sac3^20−40^. Peptides corresponding to other regions of *ct*Sac3N did not yield crystals that diffracted sufficiently well, whereas trials with longer peptides were hampered by poor solubility. The complex with *ct*Sac3^1−20^ crystallized with *P*2_1_ symmetry and the structure was determined to 1.8 Å resolution with Sac3 residues 15–20 (QVNNPF) clearly visible in the electron density, whereas the complex with *ct*Sac3^20−40^ crystallized with *P*2_1_2_1_2_1_ symmetry and the structure was determined to 1.85 Å with Sac3 residues 30–37 (SSVFGAPA) built into the model. Both crystal structures also contained the *ct*Mex67 NTF2-like domain together with *ct*Mtr2 and were refined to an *R*_free_ of 19.3% and 20.3%, respectively, with excellent geometry (Table 1). The conformation of the *ct*Mex67^NTF2^:Mtr2 heterodimer was essentially unaltered by the binding of the peptides (RMSDs compared with Mex67:Mtr2 alone of 0.50 Å over 3,758 atoms and 0.59 Å over 3,670 atoms for the Sac3^1−20^ and Sac3^20−40^ complexes, respectively; Figure S5).

**Figure 5:**
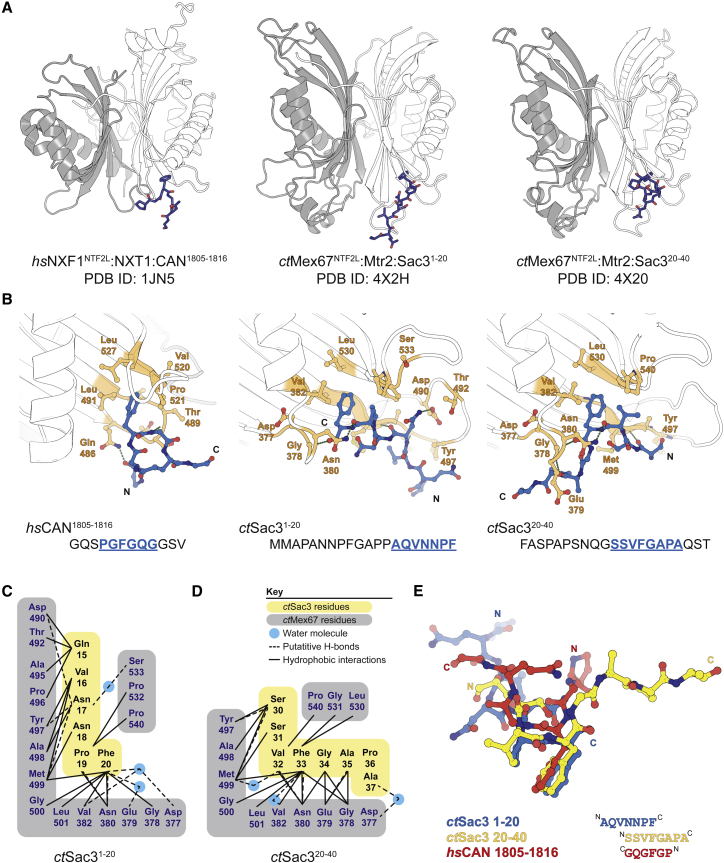
Structure of Sac3N Peptides Bound to Mex67:Mtr2 (A) Overview of the binding sites on the NTF2L domain of FG-containing peptides derived from *hs*CAN (NUP214), and from *C. thermophilum* Sac3 residues 1–20 and 20–40 (for which density was observed only for ^15^QVNNPF^20^ and ^30^SSVFGAPA^37^, respectively). The Sac3 peptides occupy the same hydrophobic pocket identified by Fribourg et al. (2001) occupied by a PGFGQ peptide derived from CAN/NUP214 in human NXF1, albeit with the chain direction reversed. (B) Details of the hydrophobic pocket in each case. The aromatic Phe side chain is central to the binding. (C and D) Schematic showing the interactions observed with the two Sac3 peptides. (E) Superposition of the two Sac3 peptides (pink: Sac3 1–20; blue: Sac3 20–40) and the CAN FG peptide (yellow). See also Figure S5.

Both crystal structures showed the Sac3N peptide bound to the same site on the Mex67 NTF2L domain (Figure 5), and the high-quality electron density maps obtained for both structures permitted an unambiguous assignment of the Sac3 residues. Both peptides bound in a similar manner, and many interactions with the NTF2L domain were conserved. Both Sac3N peptides bound in a pocket formed by the N-terminal region of α1 and the loop regions between the central β sheets of the NTF2-like fold (β3-β4 and β5-β6) that corresponded to the pocket identified by Fribourg et al. (2001) to which a PGFGQ peptide derived from nucleoporin NUP214 bound to NXF1, albeit with the chain direction reversed. This cavity was formed principally by Gly500, Leu501, Leu530, and Gly531, and accommodated a single Phe residue from either peptide (Phe20 and Phe33) with neighboring residues forming several direct and water-mediated hydrogen bonds (Figures 5C and 5D). In addition to the hydrophobic interactions dominated by the Phe side chain, putative H bonds were found between the main-chain amino group of Asn380^Mex67^ and the carbonyl group of the Sac3 Phe. Asn380 also formed putative H bonds with the residue N-terminal to the bound Phe residue (Val32 or Pro19). Conserved water-mediated putative H bonds were also present, linking Asp377^Mex67^, Val382^Mex67^, and the carbonyl of the bound Sac3N Phe. A conserved intrapeptide water-mediated H bond was seen in both Sac3N peptides, linking the Phe carbonyl to the carbonyls of Asn18 and Ser31, and the Sac3N^20−40^ peptide also contained a water-mediated H bond between the carbonyls of Gly34 and Ser31. Additional contacts were formed between Sac3N^20−40^ and the NTF2L domain, as the Phe residue bound was not at the C terminus of the peptide, with putative main-chain H bonds between Glu379^Mex67^ and Ala35^Sac3^, and a water-mediated H bond linking Asp377^Mex67^ and Ala37^Sac3^.

Although the overall fold of the *ct*Mex67 NTF2L domain or *ct*Mtr2 was not altered by the binding of either *ct*Sac3 peptide, a translation of the loop region of ∼3.0 Å prior to helix α1 of the NTF2L domain (residues 376–382, the pre-α1 loop) was observed for both peptides (Figure S5). This movement of the pre-α1 loop was not observed with a PGFGQ peptide (derived from Nup214/CAN) bound to *hs*NXF1^NTF2L^:NXT1 (PDB: 1JKG, 1JN5), possibly as a result of the *ct*Sac3 peptides forming a more intimate interaction with the NTF2L domain compared with the Nup214 peptide, which, in turn, facilitated H-bond formation between the Sac3 peptide and the pre-α1 loop. The interface formed between *hs*NXF1 NTF2L domain and the FG peptide was dominated by the aromatic side chain of the Phe residue, which contributed to 63% of the total buried surface area. However, in the *ct*Sac3 peptides in the present study, a greater contribution was made by residues neighboring the bound Phe; as a result, this residue only contributed to 39% (*ct*Sac3^1−20^) and 35% (*ct*Sac3^20−40^) of the buried surface area.

### Deleting Sac3N Produced Growth and mRNA Export Defects in *S. cerevisiae*

**Figure 6:**
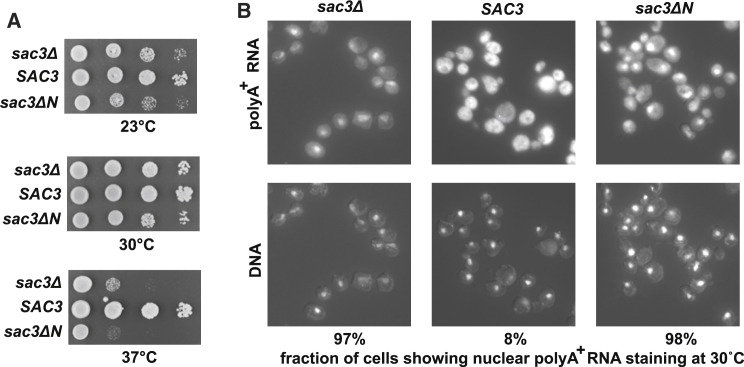
Deletion of the N Terminus of Sac3 Causes Growth and mRNA Export Defects in *S*. *cerevisiae* (A) Serial dilutions of *sac3Δ* cells carrying plasmid-borne *SAC3* (pRS314-SAC3) and *sac3* residues140–1,301 (*sac3*^140−1301^). Cells were plated on SDC-TRP plates and grown at 23°C, 30°C, and 37°C for 3 days. The deletion strain was transformed with empty pRS314 plasmid as a negative control. (B) In situ hybridization analysis of poly(A)^+^ mRNA in *sac3Δ* cells carrying plasmid-borne *SAC3* and *sac3*^140−1301^. The upper panels show poly(A)^+^ mRNA hybridized to an oligo-dT probe labeled with Cy3, whereas the lower panels show DAPI-stained DNA to identify nuclei. The fractions of cells showing nuclear accumulation of poly(A)^+^ RNA represent the means from three independent experiments in which 200 cells were scored for each. See also Figure S6.

Although not essential in yeast, Sac3 deletions show slow growth that is especially pronounced at 37°C. Deletion of the Sac3N region in *S. cerevisiae* generated a slow-growth phenotype that was indistinguishable from that observed with complete deletion of *SAC3*, and also showed a marked nuclear accumulation of poly(A) mRNA (Figure 6), indicating that the Sac3N:Mex67 interaction was required for efficient mRNA nuclear export. To exclude the possibility that the Sac3ΔN phenotypes observed were due to a complete loss of Sac3 function, the localizations of wild-type Sac3 and the mutant were compared. Both proteins were expressed and localized at the nuclear periphery (Figure S6), consistent with the phenotype observed being due specifically to the absence of the Sac3N region.

### ctMex67:Mtr2 Also Binds to FG Motifs in ctMlp1

**Figure 7:**
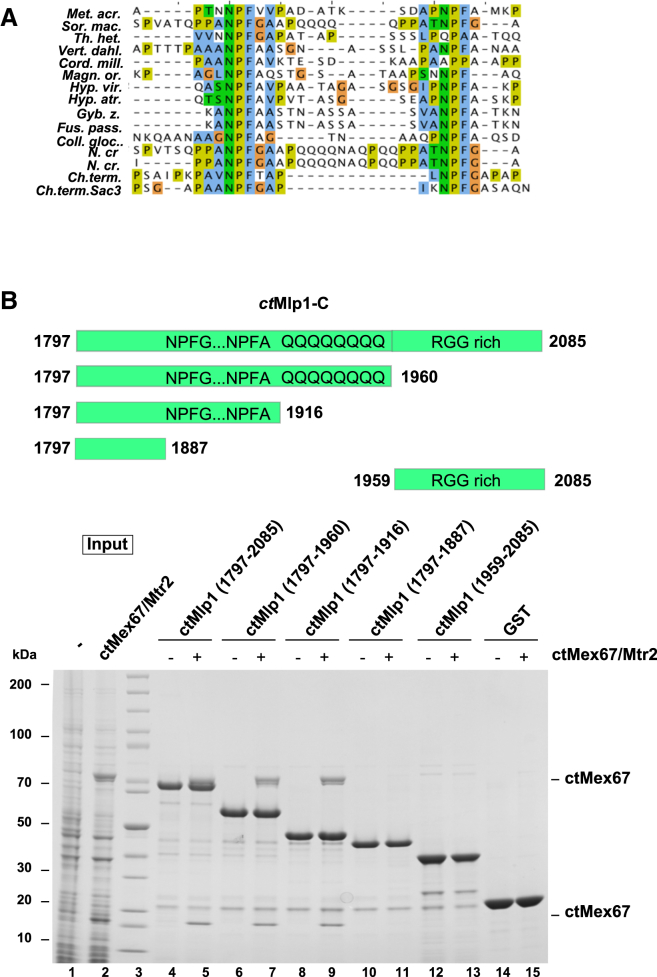
Nuclear Basket Protein *ct*Mlp1 Has Two NPFG Motifs in its C Terminus that Interact with *ct*Mex67:Mtr2 (A) The FG motif is conserved in *Pezyzomycotina* and has been aligned to two repeats of the N terminus of *ct*Sac3. (B) Different truncations of the *ct*Mlp1 C terminus (upper panel) were immobilized on GSH beads to which *E. coli* lysate containing *ct*Mex67:Mtr2 was added (lower panel). After incubation the beads were washed, eluted with SDS loading buffer, and analyzed by SDS-PAGE with Coomassie.

Because the FG-repeating sequence motifs of *ct*Sac3 interacted with *ct*Mex67:Mtr2, we investigated whether other nucleoporins might exhibit analogous interactions. The C-terminal region of the *C. thermophilum* paralog of *sc*Mlp1 or human TPR that we identified as *ct*Mlp1 (Figure 7A) had two copies of an FG motif similar to that in *ct*Sac3N. When a series of truncations from the C-terminal region of *ct*Mlp1 were immobilized on GSH beads, only those containing both FG motifs bound *ct*Mex67:Mtr2 (Figure 7B). In addition, SPR gave a binding profile very similar to that observed with Nup145, with a similar *K*_D_ (Table 2; Figure S7). The binding was weaker than that seen with Sac3N, probably because *ct*Mlp1 has only two motifs rather than the ten in *ct*Sac3N.

## Discussion

Individual components of the *C. thermophilum* TREX-2 complex have been expressed and used to obtain electron micrographs of the negatively stained complex, which indicate that it has a circular Q-like conformation (Figure 3). Class averages of these micrographs, together with the demonstration that the Sac3N region can bind to the CID region of TREX-2, are consistent with a model such as that illustrated in Figure 3F. The individual *C. thermophilum* proteins were also used to define the structural basis for the interactions between Sac3, Sus1, Cdc31, and Mex67:Mtr2. In addition to interacting with the CID region of *ct*TREX-2, the *ct*Sac3N region contains ten FG motifs separated by short linkers that bind to *ct*Mex67^NTF2L^:Mtr2, but do not bind to karyopherin family nuclear transport factors, indicating that the Sac3N motifs are distinct from those in FG nucleoporins. Structural characterization of complexes between Mex67^NTF2L^:Mtr2 and two different Sac3N peptides showed the central role of the motif's Phe residue binding into a hydrophobic pocket on Mex67^NTF2L^ in the same position where FG nucleoporins bind.

**Figure 8:**
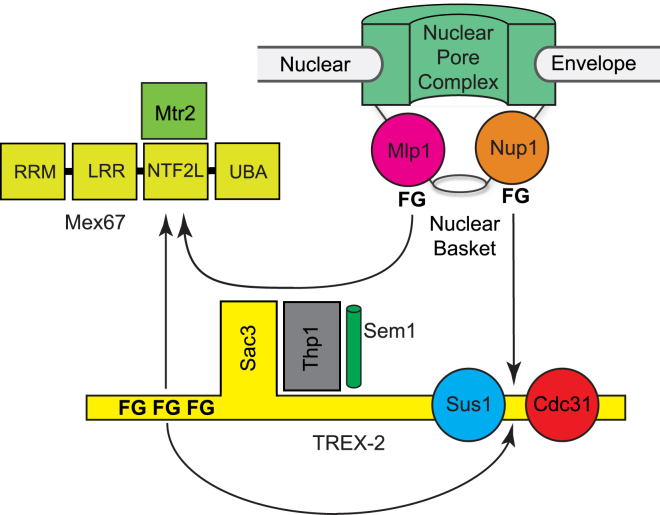
Schematic Illustration of the Interactions between TREX-2, Mex67, and NPC Components The FG motifs in Sac3N can bind to both the CID region (which contains Sus1 and Cdc31 bound to Sac3) and to the Mex67 NTF2-like domain, whereas Phe-containing motifs in nuclear basket components Mlp1 and Nup1 also bind to Mex67 and the CID region, respectively. These interactions function to localize mRNPs to the NPC nuclear face to facilitate export to the cytoplasm once processing has been completed.

Deletion of Sac3N generated growth and poly(A) mRNA export defects in *S. cerevisiae*, consistent with its functioning to facilitate efficient nuclear export by concentrating mature mRNPs close to the entrance of the NPC transport channel as a central feature of the network of interactions based on Phe-containing motifs between TREX-2, Mex67:Mtr2, and components of the nuclear basket, as illustrated schematically in Figure 8. The interaction between the CID region and Sac3N was similar to that seen with Nup1 (Jani et al., 2014), and would be consistent with a TREX-2 model in which Sac3 could adopt different conformations related to its function in integrating mRNA nuclear export with preceding steps in the gene expression pathway. An intramolecular Sac3N-Sac3^CID^ interaction would be consistent with the appearance of annuli on electron micrographs that could come about if TREX-2 were to adopt a conformation like that illustrated schematically in Figure 3F. Although further work will be necessary to evaluate the precise role of the Sac3N region, the intermolecular Sac3N:Sac3^CID^ interaction could control TREX-2 interactions by generating an autoinhibitory conformation whereby it was unable to interact with either proteins such as Nup1 and Mlp1 in the nuclear basket or with Mex67:Mtr2. For example, when bound to the CID region, the Sac3N region would probably not be able to also bind Mex67:Mtr2, whereas binding of Mex67:Mtr2 to Sac3N would free the CID region to bind to nucleoporins such as Nup1 and so mediate association of TREX-2 with NPCs. The TREX-2 mediated concentration of mature mRNPs at the nuclear entrance of the NPC transport channel (Ellisdon et al., 2012) appeared to be complemented by the nuclear basket protein, Mlp1, which contained FG sequence motifs and which interacted in a manner analogous to Mex67:Mtr2. In humans, TPR (the Mlp1 homolog) contributes to the localization of TREX-2 to the nuclear pore (Wickramasinghe et al., 2014). Moreover, in yeast, the Mlp1 C-terminal region also interacts with Nab2, an mRNP component that binds to the poly(A) tails of transcripts (Green et al., 2003; Grant et al., 2008). Here, we have shown that both TREX-2 and Mlp1 appear to bind to Mex67:Mtr2 using similar Phe-rich motifs. Although in vitro the binding constant for binding Mlp1 was only ∼300 nM, the NPC nuclear basket contains at least 16 copies of Mlp1 (Ori et al., 2013), so the high local concentration of the Mlp1 FG motif would probably generate a higher avidity for Mex67:Mtr2 that could contribute to increasing the local concentration of mRNPs at the nuclear basket at the entrance to the NPC transport channel and complement the function of TREX-2, which also binds to both NPCs and mRNPs (Ellisdon et al., 2012; Jani et al., 2014). Although further work will be necessary to define their precise temporal sequence, the interactions between Mex67:Mtr2 and the FG-containing repeats in Sac3N, Mlp1, and the nucleoporins within the pore transport channel would facilitate the TREX-2 mediated transfer of mature mRNAs from the pre-mRNA processing machinery to NPCs for export to the cytoplasm. Moreover, the directionality of movement through this series of interactions would be facilitated by the FG nucleoporins binding to the Mex67 UBA domain in addition to NTF2L domain used by the other components.

In summary, a combination of structural, biochemical, and cellular methods has been used to characterize the *C. thermophilum* TREX-2 complex and explore its interactions with other components of the gene expression pathway. The Sac3N region, which contains tandem repeats of an FG sequence motif, binds to the bulk mRNA nuclear export factor, Mex67:Mtr2; importantly, deletion of this region of Sac3 results in both mRNA export and growth defects. Moreover, both electron microscopy and solution studies indicate that the *C. thermophilum* TREX-2 complex has a potential to generate annular particles through an interaction between the Sac3N and CID regions that could affect the interactions of TREX-2 with other components of the nuclear gene expression pathway. Moreover, both the NPC nuclear basket components Mlp1 and the Sac3 N-terminal domain in TREX-2 bind to Mex67:Mtr2, consistent with their function as an interaction platform to concentrate mature mRNPs at the entrance to the NPC transport channel and thus facilitate their nuclear export.

## Experimental Procedures

### Protein Expression, Purification, and In Vitro Reconstitution

Constructs corresponding to *THP1*, *SAC3*, *SEM1*, *CDC31*, *SUS1*, *KAP104*, *CRM1*, *NUP145*, *MEX67*, and *MTR2* were amplified by PCR from *C*. *thermophilum* cDNA or gDNA. Plasmid details are provided in Table S1. Proteins were expressed in *E. coli* BL21 (DE3) CodonPlus-RIL using isopropyl β-D-1-thiogalactopyranoside induction at 18°C for 16 hr. Cells were lysed in 50 mM Tris (pH 7.4), 500 mM NaCl, and 20 mM imidazole (pH 8.0). The lysate was clarified by centrifugation and the supernatant was incubated with Ni-NTA beads (Qiagen) for 1 hr. The beads were pelleted by gravity flow filtration and washed with 60 ml of 50 mM Tris (pH 7.4), 500 mM NaCl, and 20 mM imidazole (pH 8.0). The protein was eluted in 10 ml of the same buffer containing 250 mM imidazole (pH 8.0). The His_6_ tag was removed by overnight incubation with 1 mg of His_6_-TEV protease at 4°C.

Individual proteins and complexes were purified to homogeneity by gel-filtration chromatography in 20 mM Tris-HCl (pH 7.4) and 200 mM NaCl using a Superdex200 column (GE Healthcare). Peak fractions containing pure and stoichiometric protein complexes were pooled and concentrated, flash-frozen in liquid nitrogen, and stored at −80°C until required. Synthetic peptides were purchased from Designer BioScience at 95% purity and were dissolved in Milli-Q water (Millipore) prior to use. The *ct*Mex67-NTF2L^NTF2L^:*ct*Mtr2:*ct*Sac3 complexes were prepared by mixing *ct*Sac3 peptides and purified *ct*Mex67-NTF2L^NTF2L^:*ct*Mtr2 at a 5:1 molar ratio. The mixture was centrifuged for 20 min at 4°C prior to setting up crystallization trials.

For in vitro reconstitution of the TREX-2 complex, the yeast strain DS1-2b (Nissan et al., 2002) was co-transformed with YEplac112 (TRP1)-pGal1-10-pA-TEV-Sac3 (1–1,191) (Leu2d) and YEplac195 (URA1)-Cdc31-pGal1-10-Sus1. Yeast cells were grown in SRC-Leu-Ura medium to an OD_600_ of 2.5–3, after which they were shifted to YPG medium and induced for 6 hr. Cells were harvested in 400 mM NaCl, 20 mM Na-HEPES, 2 mM MgCl_2_, 0.01% NP-40, and 5% glycerol supplemented with FY complete protease inhibitor (Serva) before lysing in the same buffer using a Retsch cryo mill MM400 (Retsch) and the cleared lysates incubated with IgG-Sepharose 6 fast-flow beads (GE Healthcare) overnight at 4°C on a rotating wheel. After washing, the beads were incubated with Thp1:Sem1 complex that had been purified using Ni-affinity chromatography. After 30 min incubation and washing, TEV cleavage was carried out at 16°C for 2 hr. The TEV eluate was then incubated with Anti-Flag M2 affinity gel (Sigma) at 4°C for 1 hr, after which the complex was eluted using 1× Flag peptide (Sigma) for 1 hr at 4°C.

### DID Labeling

GST-TEV-*ct*Sac3^1−1191^-DID1, *ct*Sus1, and *ct*Cdc31 were co-expressed in *E. coli* at 30°C for 3 hr. After lysis, the complex was immobilized on GSH beads. The Ni eluate of the *ct*Thp1:Sem1 was added in excess to the GSH beads and incubated for 1 hr. After washing, Dyn2 and DID2-flag were added and the complex was incubated at 4°C overnight (Flemming et al., 2010). After washing, TEV cleavage was performed for 2 hr at 16°C followed by an affinity step on anti-FLAG M2 agarose and elution with FLAG peptide.

### Pull-Down Assays

Proteins for pull-down assays were purified in 200 mM or 400 mM NaCl, 20 mM Na-HEPES (pH 7.5), 2 mM MgCl_2_, 5% glycerol, and 0.01% NP-40. Baits were immobilized on GSH beads. *E. coli* lysates from cells expressing the proteins of interest or *E. coli* lysates mixed with pre-purified bait proteins were added so that the protein of interest was in 5-fold excess to the bait protein. After incubation on a turning wheel at 4°C and extensive washing, the proteins were eluted by heating at 90°C for 2 min in 200 mM Tris-HCl (pH 6.8), 8% SDS (w/v), 40% glycerol, 0.4% bromophenol blue, and 100 mM DTT, and analyzed by SDS-PAGE with Coomassie staining.

### Crystallography

Crystals of *ct*Mex67^NTF2L^:*ct*Mtr2:*ct*Sac3 complexes were grown at 18°C by sitting-drop vapor diffusion using sparse-matrix screens. *ct*Mex67^NTF2L^:*ct*Mtr2:*ct*Sac3^1−20^ was crystallized by mixing 200 nl of protein complex with 200 nl of 30% PEG 400, 0.1 M NaCl, 0.1 M MgCl_2_, and 0.1 M Na citrate (pH 5.5), whereas Mex67^NTF2L^:Mtr2:Sac3^20−40^ was crystallized by mixing 200 nl of protein complex with 200 nl of 20% PEG 6000, 1 M LiCl, and 0.1 M 2-(*N*-morpholino)ethanesulfonic acid (pH 6.0). Plate-like crystals of *ct*Sac3^1086−1,170^:*ct*Sus1:*ct*Cdc31 complex were grown in 20% PEG 3350 and 0.1 M Na succinate by sitting-drop vapor diffusion. Crystals were harvested and cryo-cooled in mother liquor supplemented with 20% glycerol. Crystallographic data were collected at the European Synchrotron Radiation Facility (Grenoble, France) using beamlines ID14-4 and ID23-1, indexed and integrated using X-ray diffraction (Kabsch, 2010), and reflections merged and scaled using AIMLESS (Collaborative Computational Project, Number 4, 1994). The structures were solved by molecular replacement using PHASER in the PHENIX suite (Adams et al., 2010) using PDB: 3FWB and 4WP5 as search models. Iterative building and refinement was conducted using Coot (Emsley and Cowtan, 2004) together with PHENIX (Adams et al., 2010) and BUSTER-TNT (Bricogne et al.,, 2011), resulting in a structure with *R*_work_/*R*_free_ = 17.1/19.3 for *ct*Mex67^NTF2L^:*ct*Mtr2:*ct*Sac3^1−20^, *R*_work_/*R*_free_ = 17.4/21.5 for *ct*Mex67^NTF2L^:*ct*Mtr2:*ct*Sac3^20−40^, and *R*_work_/*R*_free_ = 23.2/28.7 for *ct*Sac3^1086−1,170^:*ct*Sus1:*ct*Cdc31.

### Two-Dimensional Electron Microscopy and Image Processing

For electron microscopy, 200 μl of affinity-purified TREX-2 complex, eluted using 1× FLAG peptide as described above, was applied onto 200 μl of 7.5% (v/v) glycerol cushion. The cushion was layered on a 10%–30% (v/v) linear glycerol gradient in 400 mM NaCl, 20 mM Na-HEPES (pH 7.5), and 2 mM MgCl_2_. Samples were centrifuged in an SW60 Ti Rotor (Beckman Coulter) for 18 hr at 50,000 rpm at 4°C, followed by fractionation.

Electron micrographs of negatively stained TREX-2 were obtained as described (Lutzmann et al., 2005). 5 μl of samples of TREX-2 in 400 mM NaCl, 20 mM Na-HEPES (pH 7.5), and 2 mM MgCl_2_ were applied to freshly glow-discharged carbon-coated grids for 2 min, washed three times with water, stained with 2% (w/v) uranyl formate, and dried. Micrographs were taken using Tecnai F20 electron microscope (FEI) operating at 200 kV with an Eagle bottom-mounted 4K, HS CCD camera (Tecnai) at a nominal magnification of 50,000×. 27,189 particles were selected manually using the interactive program BOXER (Ludtke et al., 1999), and image processing was carried out using the IMAGIC-4D package (van Heel et al., 1996). Particles were band-pass filtered and normalized in their gray value distribution and mass-centered. Two-dimensional alignment, classification, and iterative refinement of class averages were performed as described previously (Liu and Wang, 2011).

Electron micrographs of DID-labeled TREX-2 complex negatively stained with uranyl acetate (2% w/v) were obtained at 80 kV using a JEOL JEM-1400 microscope under low-dose conditions and were recorded on a 2 K × 2 K Tietz-CCD camera (TVIPS F224) at a nominal magnification of 20,000×. BOXER was used to select manually 589 single particle images of the labeled complex that were then imported into the IMAGIC 5 package.

### Biophysical Methods

SPR measurements were made using a BIAcore T200 instrument (GE Healthcare) at a flow rate of 30 μl/min in 20 mM Na-HEPES (pH 7.5), 400 mM NaCl, 2 mM MgCl_2_, 5% glycerol, and 0.01% NP-40 at 25°C. GST-tagged *ct*Sac3N^(1−210)^, *ct*Nup145^(213−567)^, *ct*Mlp1^(1797−1960)^ or GST were captured by an anti-GST antibody-coated CM5 sensor chip (GE Healthcare) prepared according to the supplier’s instructions. A series of concentrations of *ct*Mex67:Mtr2 (0.11, 0.33, 1.1, 3.3, 10 μM) was injected for 120 s and dissociation monitored for 600 s. The sensor surface was regenerated after each injection with a 1-min injection of 10 mM glycine (pH 2.1).

## Author Contributions

L.D. identified the *C. thermophilum* TREX-2 components and assayed their interactions; D.F. obtained the electron microscopy data; E.V., S.A., and M.S. obtained the crystal structures; S.H.McL. performed SPR assays; all authors interpreted the data and wrote the manuscript.

## Figures and Tables

**Table 1:** Crystallographic Data

	*ct*Mex67^NTF2L^:*ct*Mtr2:*ct*Sac3^(1−20)^	*ct*Mex67^NTF2L^:*ct*Mtr2:*ct*Sac3^(20−40)^	*ct*Sac3^CID^:*ct*Sus1:*ct*Cdc31
**Data Collection Statistics**			

Wavelength (Å)	0.9763	0.9763	0.9700
Space group	*P* 2_1_	*P* 2_1_ 2_1_ 2_1_	*P* 2_1_
Unit cell: *a*, *b*, *c* (Å); α, β, γ (°)	53.0, 74.2, 54.8; 90.0, 113, 90.0	56.7, 73.0, 93.2; 90.0, 90.0, 90.0	56.5, 168.3, 69.2; 90.0, 112.2, 90.0
Resolution range (Å)^a^	44.9–1.80 (1.84–1.80)	48.5–1.85 (1.89–1.85)	19.9–3.31 (3.58–3.31)
Unique reflections	36,313	33,498	17,487
Total observations	251,511	152,415	46,303
<*I*/σ(*I*)>^a^	9.3 (1.8)	15.5 (1.9)	6.9 (2.2)
*R*_p.i.m._^a^	0.060 (0.45)	0.047 (0.37)	0.13 (0.49)
Completeness (%)^a^	99.9 (99.6)	99.3 (93.7)	98.0 (99.0)
Multiplicity	6.9	4.5	2.6
Wilson B factor	16.7	24.3	78.4

**Refinement Statistics**			

*R*_work_/*R*_free_ (%)	16.1/19.3	16.7/20.3	21.8/27.7
Non-hydrogen atoms	3,306	3,167	5,952
Water molecules	325	275	0
Bond length RMSD (Å)	0.006	0.008	0.004
Bond angle RMSD (°)	1.014	1.11	0.792
Ramachandran favored/outliers (%)	98.6/0	98.0/0	98.5/0
MolProbity score/percentile	0.87 (100th percentile)	1.01 (100th percentile)	1.07 (100th percentile)

a Highest-resolution shell in parentheses.

**Table 2:** Summary of the Surface Plasmon Resonance Measurements for the Binding of *ct*Mex67:Mtr2 to GST-Sac3N, GST-Nup145 and GST-Mlp1

Ligand	*k*_off1_ (s^−1^)	*k*_off2_ (s^−1^)	*k*_on1_ (M^−1^ s^−1^)	*k*_on2_ (M^−1^ s^−1^)	*K*_D1_ (nM)	*K*_D2_ (nM)
GST-Sac3	0.073 ± 0.007	4.9 ± 0.3 × 10^−3^	1.9 ± 0.7 × 10^6^	3.3 ± 1.1 × 10^4^	39 ± 15	148 ± 50
GST-Nup145	0.071 ± 0.004	7.4 ± 0.2 × 10^−3^	3.0 ± 0.5 × 10^5^	2.8 ± 0.8 × 10^4^	238 ± 42	264 ± 77
GST-Mlp1	0.071 ± 0.002	8.8 ± 1.1 × 10^−3^	2.3 ± 0.4 × 10^5^	2.1 ± 0.6 × 10^4^	309 ± 54	419 ± 123

## Accession Numbers

The accession numbers for the coordinates and structure factors for the crystals structures of *ct*Mex67^NTF2L^:*ct*Mtr2:*ct*Sac3^1–20^, *ct*Mex67^NTF2L^:*ct*Mtr2:*ct*Sac3^20–40^, and *ct*Sac3^1086–1170^:*ct*Sus1:*ct*Cdc31 reported in this paper are PDB: 4X2H, 4X2O, and 4WPX, respectively.

## Supplemental Information

Document S1. Supplemental Experimental Procedures, Table S1, and Figures S1–S7

Document S2. Article plus Supplemental Information
