# Supplementary material for: Structural Characterization of the Chaetomium thermophilum TREX-2 Complex and its Interaction with the mRNA Nuclear Export Factor Mex67:Mtr2
Source: Structure. 2015 Jul 7;23(7):1246–57. doi: 10.1016/j.str.2015.05.002 (PMC4509546; doi:10.1016/j.str.2015.05.002)
Supplement: Document S1. Supplemental Experimental Procedures, Table S1, and Figures S1–S7 [file mmc1.pdf]

Structure, Volume *23*

Supplemental Information

Structural Characterization of the *Chaetomium thermophilum* TREX-2 Complex and its Interaction with the mRNA Nuclear Export Factor Mex67:Mtr2

Lyudmila Dimitrova, Eugene Valkov, Shintaro Aibara, Dirk Flemming, Stephen H. McLaughlin, Ed Hurt, and Murray Stewart

SUPPLEMENTARY MATERIAL

Structural Characterization of the *Chaetomium thermophilum* TREX-2 Complex and its Interaction with the mRNA Nuclear Export Factor Mex67:Mtr2

Lyudmila Dimitrova[1,2], Eugene Valkov[1], Shintaro Aibara[1], Dirk Flemming[2], Stephen H. McLaughlin[1], Ed Hurt[2] and Murray Stewart[1]

[1]MRC Laboratory of Molecular Biology, Francis Crick Ave., Cambridge CB2 0QH, UK and [2] Biochemie-Zentrum der Universität Heidelberg, INF328, D-69120 Heidelberg, Germany.

Supplementary Experimental Procedures

Strains and plasmids: *ctTHP1* was cloned into the BamHI and EcoRI sites of pET-Duet-1 (Novagen) in frame with the His_6 tag, with *ctSEM1* cloned in the second multiple cloning site (MCS) via NdeI/XhoI. *ctSAC3-*$^{M415-755}$ together with an additional ribosomal binding site was cloned into EcoRI/Pst1 sites of pET-Duet-1-HIS_6-*ctTHP1-ctSEM1*. *ctSUS1* was cloned into BamHI/SacI in frame with a HIS_6 tag, whereas *ctCDC31* was cloned between the NdeI and XhoI sites in the second MCS. *ctSAC3-CID*$^{1085-1170}$ was inserted between NdeI and BamHI sites of pET24d-GST-TEV. For TREX-2, reconstitution *ctSAC3*$^{1-1191}$-FLAG was inserted at the NdeI/BamHI sites of YEplac112(TRP1)-pGal1-10-pA-TEV(Leu2d). His_6-*ctSUS1* was cloned in the P1 site of YEplac195(URA1)-P2-pGal1-10-P1 and *ctCDC31* was cloned in the P2 site. For pull-down assays, *ctMLP1-C* and truncations, *ctSAC3*$^{1-755}$ and truncations, Nup145$^{213-567}$, *ctCRM1*, *ctKAP104* were cloned into the NdeI/BamHI sites of pET24d-GST-TEV. *ctMEX67* truncations and *ctMTR2* were cloned into the BamHI/EcoRI sites and NdeI/XhoI sites of pET-Duet-1, respectively. For the growth and mRNA export assays *sac3Δ(*G1) strain was transformed with pRS314(TRP), pRS314-Sac3 and pRS314-Sac3$^{140-1301}$. To check the localization of wt and mutant Sac3, GFP was inserted at a BamHI site at the Sac3 N-terminus.

Growth Assays Cells were freshly plated overnight on SDC-TRP plates. On the following day, they were diluted to OD_{600} = 1 after which they were spotted in 10 fold dilutions on SDC-TRP and grown for 3 days at 23°C, 30°C and 37°C.

Analysis of mRNA nuclear export by in situ hybridization and fluorescent microscopy GFP labelled SAC3 and mutant were grown in selective media at 30˚C to logarithmic growth. For the *in situ* hybridization cells were grown at 30˚C at selective media before being fixed. Analysis of poly(A)$^+$ RNA export by *in situ* hybridization was carried out using Cy3-labeled oligo(dT) probes essentially as described in Amberg et al. (1992). For fluorescence microscopy, an Imager Z1 (Carl Zeiss) with a 63× NA 1.4 plan apochromat oil immersion lens (Carl Zeiss) and DICIII, 4′,6-diamidino-2-phenylindole, HECy3 or HEeGFP filter sets were used. Images were acquired with an AxioCamMRm camera (Carl Zeiss) and AxioVision 4.3 (Carl Zeiss) at resolution 1,388 × 1,040 (Binning 1×1, gain factor 1).

Table S1. Plasmids and yeast strains, related to Experimental Procedures

Plasmids	Origin
pET-Duet-1-*HIS6-ctTHP1-ctSEM1*	This study
pET-Duet-1-*HIS6-ctTHP1-ctSAC3M(415-755)-ctSEM1*	This study
pET-Duet-1-*HIS6-ctSUS1-ctCDC31*	This study
pET24d-*GST-TEV- ctSAC3-CID*(1085-1170)	This study
YEplac112 (TRP1)-*pGAL1-10-pA-TEV-ctSac3(1-1191)-FLAG*	This study
YEplac195(URA1)-*ctCDC31-pGAL1-10-HIS6-ctSUS1*	This study
pET24d-*GST-TEV- ctSAC3*(1-784)	This study
pET24d-*GST-TEV- ctSAC3*(1-112)	This study
pET24d-*GST-TEV- ctSAC3*(1-112)	This study
pET24d-*GST-TEV- ctSAC3*(21-784)	This study
pET24d-*GST-TEV- ctSAC3*(44-784)	This study
pET24d-*GST-TEV- ctSAC3*(105-784)	This study
pET24d-GST-TEV- *ctNUP145(213-567*)	This study
pET24d-GST-TEV- *ctKAP104*	Kressler et al. (2012)
pET24d-GST-TEV- *ctCRM1*	Monecke et al. (2013)
pETDuet-1- *HIS6-ctMEX67()-ctMTR2*	This study
pETDuet-1-*HIS6-TEV-ctMEX67(NTF2L)-ctMTR2*	This study
pETDuet-1-*HIS6-MEX67(UBA)*	This study
pET24d-HIS6-TEV- ctSac3(44-94)	This study
pET24d-*GST-TEV- ctMLP1*(1797-2085)	This study
pET24d-GST-TEV- *ctMLP1*(1797-1960)	This study
pET24d-GST-TEV- *ctMLP1*(1797-1916)	This study
pET24d-GST-TEV- *ctMLP1*(1797-1887)	This study
pET24d-GST-TEV- *ctMLP1*(1959-2085)	This study
pRS314(TRP)	Sikorski and Hieter (1989)
pRS314-*SAC3*	Ellisdon et al. (2012)
pRS314-*SAC3* (140-1301)	This study
pRS314-*GFP-SAC3*	This study
pRS314-*GFP-SAC3* (140-1301)	This study

Strains	Origin
DS1-2b	Nissan et al. (2002)
sac3Δ(G1)	Fischer et al. (2002)

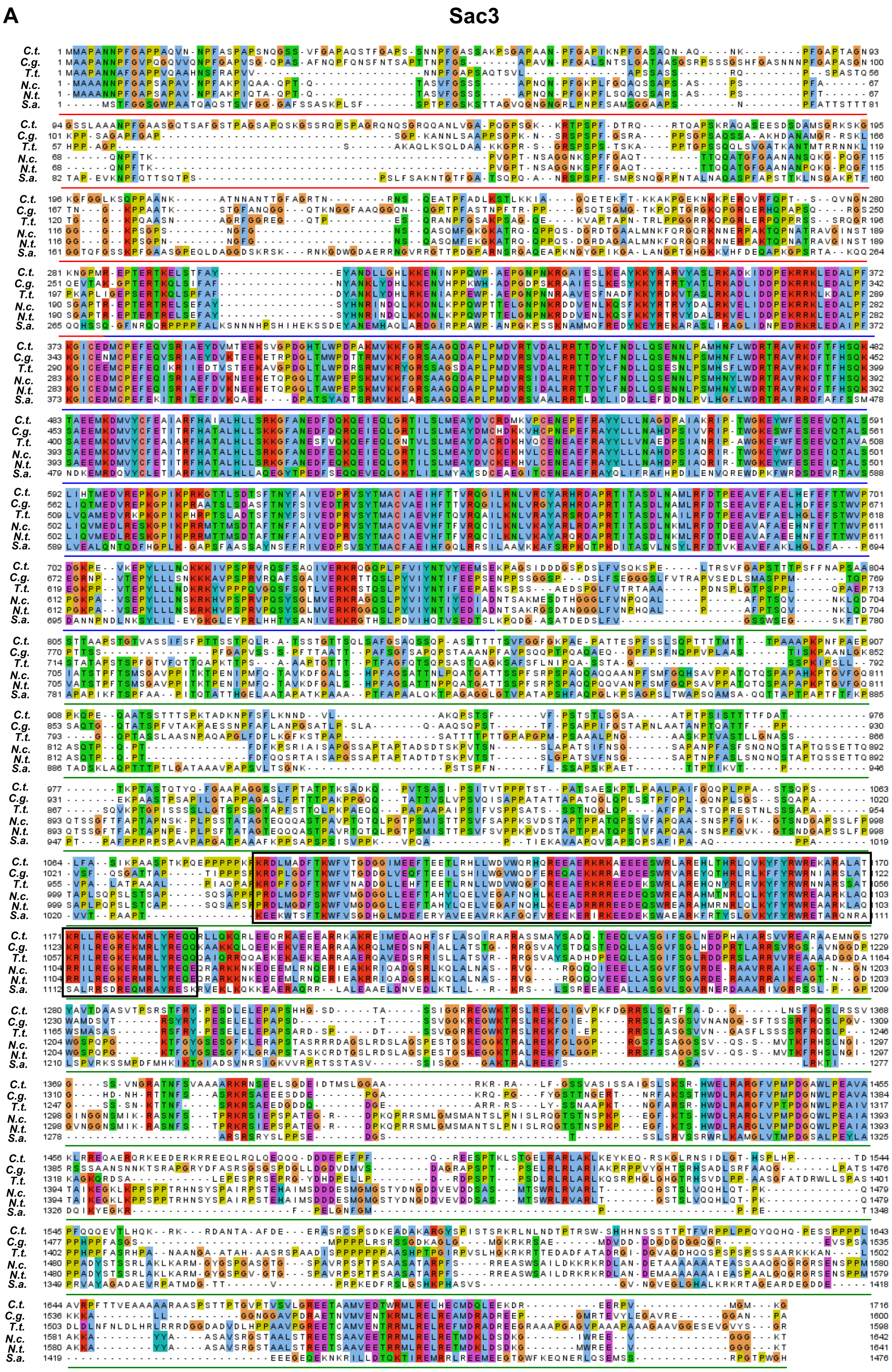

Supplementary Figure S1A, related to Figure 1: (**A**) Multiple sequence alignment of Sac3 (*Chaetomium thermophilum (C.t.)*, *Chaetomium globosum (C.g.)*, *Thielavia terrestris (T.t.)*, *Neurospora crassa (N.c.)*, *Nurospora tetrasperma (N.t.)* and *Scedosporium apiospermum (S.a.)*.

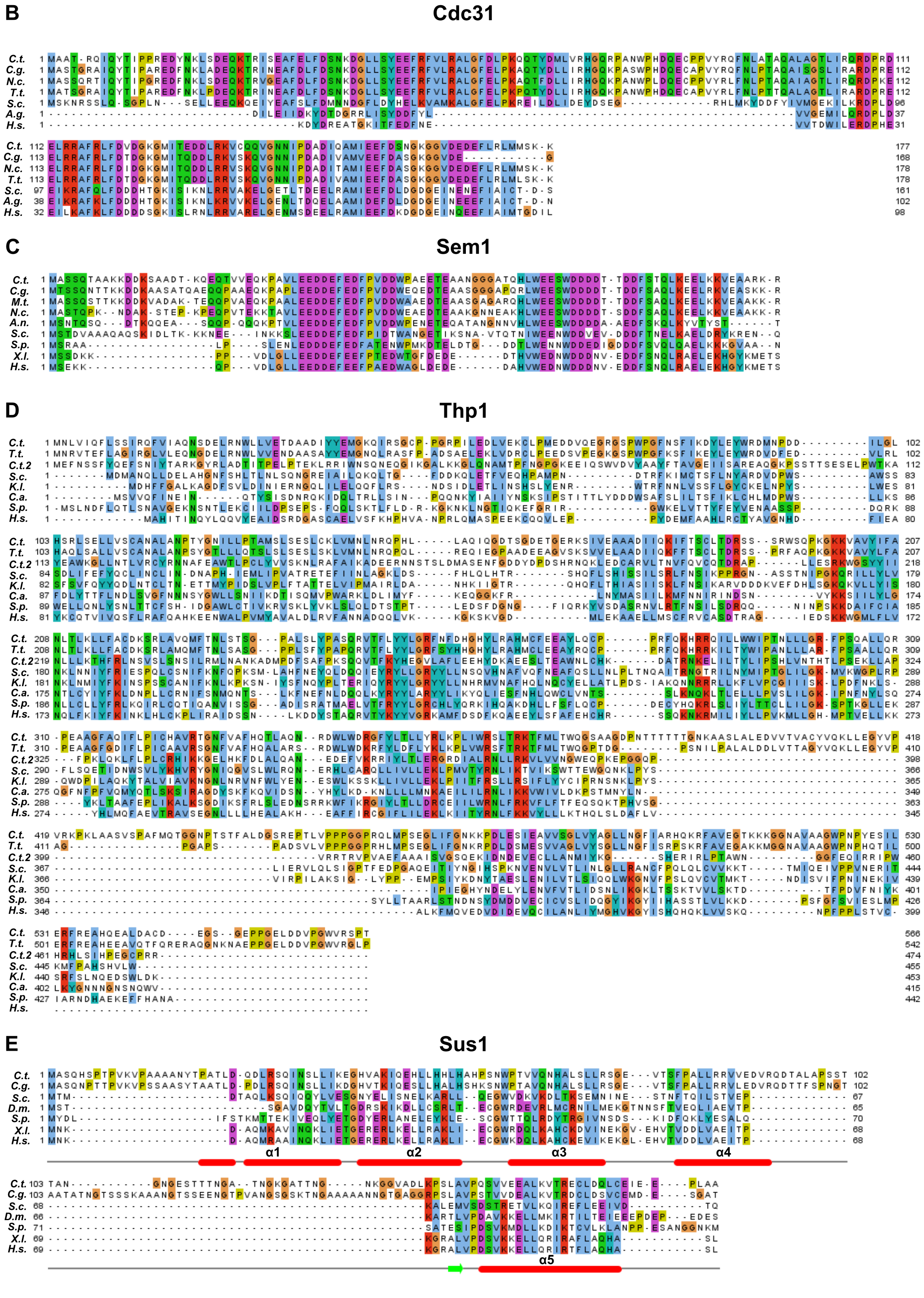

Supplementary Figure S1 B-E, related to Figure 1: Multiple sequence alignments for Cdc31 (**B**), Sem1 (**C**),Thp1 (**D**) and Sus1 (**E**) from *C. thermophilum (C.t.)*, *T. terrestris (T.t.)*, *C. thermophilum2* (CTHT_0056910) (*C.t.2*), *Chaetomium globosum (C.g.), S. cerevisiae (S.c.)*, *K. lactis (K.l.)*, *C. albicans (C.a.)*, *S. pombe (S.p.)*, *Homo sapien (H.s.), Drosophila melanogaster (D.m.), Xenopus laevis (X.l.)*, *Neurospora crassa (N.c.)* and *Aspergillus nidulans (A.n.)*.

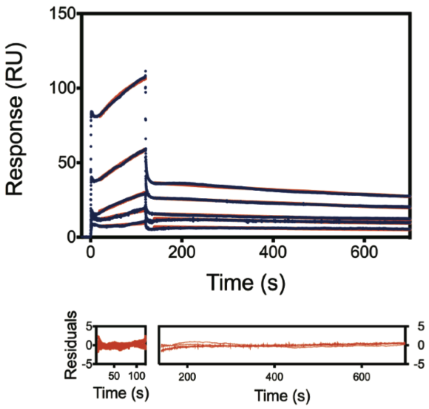

Supplementary Figure S2, related to Figure 2. Binding of the Sac3^{CID}:Cdc31:Sus1 complex to the Sac3 N-terminus analyzed by Surface Plasmon Resonance. Both the on- and off-rate constants were very slow (k_a = 235 M^{-1} s^{-1} and k_d = 5.2 x 10^{-4} s^{-1} with the overall equilibrium constant K_d equating to 2.2 μM).

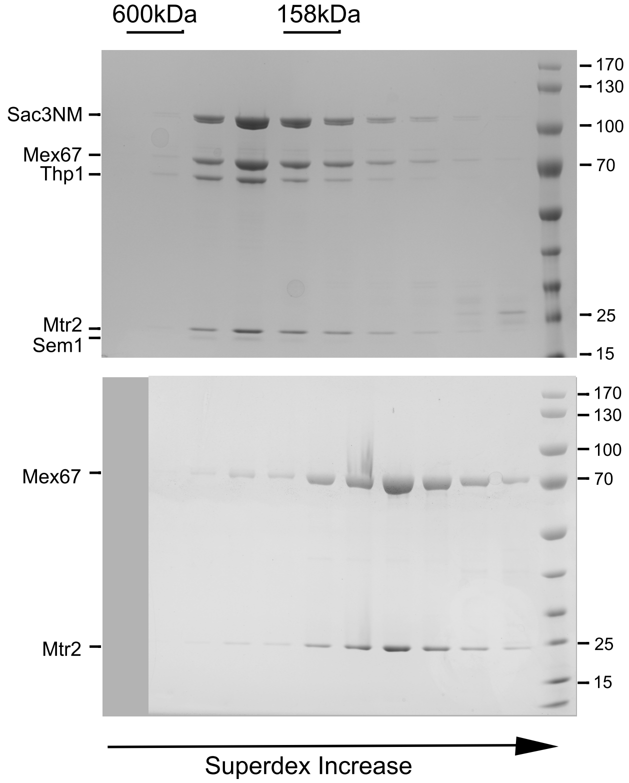

Supplementary Figure S3, related to Figure 4. Comparison of the size exclusion chromatography of the *ct*Sac3NM:Thp1:Sem1:Mex67:Mtr2 pentameric complex and the *ct*Mex67:Mtr2 complex. The *in vitro* reconstituted pentameric complex and *ct*Mex67:Mtr2 were loaded onto a Superdex 200 increase size exclusion column. The fractions were analysed by SDS-PAGE and stained by Coommassie.

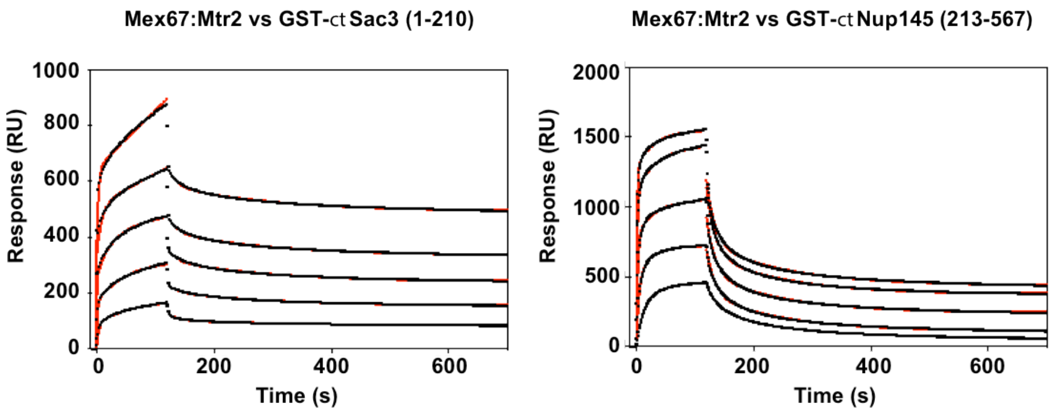

Kd1(nM)= 39+/-15
Kd2(nM)= 148+/-50

Kd1(nM)= 238+/-42
Kd2(nM)= 264+/-77

Supplementary Figure S4, related to Table 2 and Figure 5. SPR analysis of the binding of *ct*Mex67:Mtr2 to *ct*Sac3 (1-210) and *ct*Nup145N. The data were fitted to a two-phase model with K_d1 and K_d2 depicted under the sensograms.

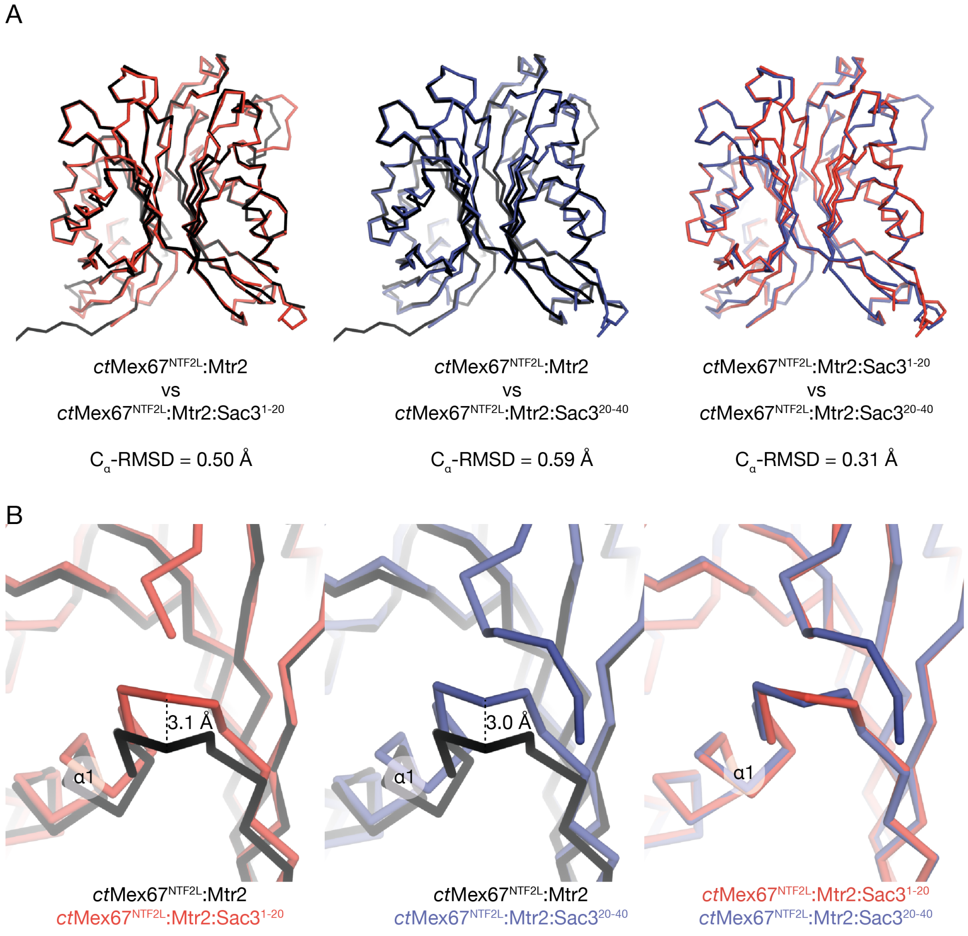

Supplementary Figure S5, related to Figure 5. Conformational changes in the pre-α1 loop of Mex67^{NTF2L} on binding different peptides. Whereas binding of either Sac3$^{1-20}$ or Sac3$^{20-40}$ produced a ~3 Å shift in the pre-α1 loop, binding a FG peptide produced little change.

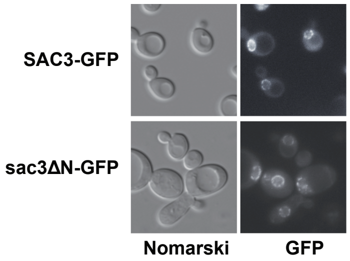

Supplementary Figure S6, related to Figure 6. Deletion of Sac3N residues 1-136 does not inhibit Sac3-GFP binding to NPCs. Both SAC3-GFP (pRS315-GFP-SAC3) and Sac3 (140-1301)-GFP shown nuclear rim staining in *S. cerevisiae* cells.

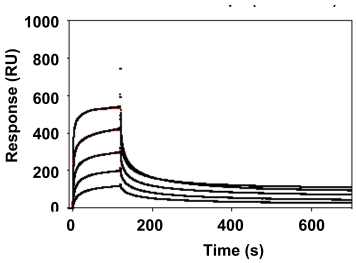

Supplementary Figure S7, related to Table 2 and Figure 7. SPR analysis of the binding of *ct*Mlp1 NPFG motifs to *ct*Mex67:Mtr2.

SUPPLEMENTARY REFERENCES

Amberg, D.C., Goldstein, A.L. and Cole, C.N. 1992. Isolation and characterization of RAT1: an essential gene of *Saccharomyces cerevisiae* required for the efficient nucleocytoplasmic trafficking of mRNA. Genes Dev. *6*, 1173-1189.

Ellisdon, A.M., Dimitrova, L., Hurt, E. and Stewart, M. 2012. Structural basis for the assembly and nucleic acid binding of the TREX-2 transcription-export complex. Nature Struct. Mol. Biol. *19*, 328-336.

Fischer, T., Strässer, K., Rácz, A., Rodriguez-Navarro, S., Oppizzi, M., Ihrig, P., Lechner, J. and Hurt, E. 2002. The mRNA export machinery requires the novel Sac3p-Thp1p complex to dock at the nucleoplasmic entrance of the nuclear pores. EMBO J. *21*, 5843-5852.

Kressler, D., Bange, G., Ogawa, Y., Stjepanovic, G., Bradatsch, B., Pratte, D., Amlacher, S., Strauß, D., Yoneda, Y., Katahira, J., Sinning, I. and Hurt, E. 2012. Synchronizing nuclear import of ribosomal proteins with ribosome assembly. Science. *338*, 666-671.

Monecke, T., Haselbach, D., Voß, B., Russek, A., Neumann, P., Thomson, E., Hurt, E., Zachariae, U., Stark, H., Grubmüller, H., Dickmanns, A. and Ficner, R. 2013. Structural basis for cooperativity of CRM1 export complex formation. Proc. Natl. Acad. Sci., USA. *110*, 960-965.

Nissan, T.A., Bassler, J., Petfalski, E., Tollervey, D. and Hurt, E. 2002. 60S pre-ribosome formation viewed from assembly in the nucleolus until export to the cytoplasm. EMBO J. *21*, 5539-5547.

Sikorski, R.S. and Hieter, P. 1989. A system of shuttle vectors and yeast host strains designed for efficient manipulation of DNA in *Saccharomyces cerevisiae*. Genetics, *122*, 19-27.
